# Adherencia a la guía de práctica clínica para el manejo hospitalario de pacientes con insuficiencia cardíaca descompensada en una unidad de cuidados coronarios de Colombia

**DOI:** 10.47487/apcyccv.v4i4.330

**Published:** 2023-12-27

**Authors:** Juan José Diaztagle Fernández, Diana Vargas Vergara, Carlos Alfonso Madariaga Carocci, David Hamon-Rugeles, Juan Pablo Castañeda-González

**Affiliations:** 1 Departamento de Medicina Interna, Fundación Universitaria de Ciencias de la Salud, Hospital de San José, Bogotá, Colombia. Departamento de Medicina Interna Fundación Universitaria de Ciencias de la Salud Hospital de San José Bogotá Colombia; 2 Departamento de Cardiología, Fundación Universitaria de Ciencias de la Salud, Hospital de San José, Bogotá, Colombia. Departamento de Cardiología Fundación Universitaria de Ciencias de la Salud Hospital de San José Bogotá Colombia; 3 Instituto de Investigaciones, Fundación Universitaria de Ciencias de la Salud, Hospital de San José, Bogotá, Colombia. Instituto de Investigaciones Fundación Universitaria de Ciencias de la Salud Hospital de San José

**Keywords:** Insuficiencia Cardiaca, Cumplimiento de la Medicación, Unidades de Cuidados Coronarios, Guía de Práctica Clínica, Heart Failure, Medication Adherence, Coronary Care Units, Clinical Practice Guidelines

## Abstract

**Objetivo:**

. Evaluar la adherencia a las recomendaciones para el diagnóstico y manejo emitidas por la Sociedad Europea de Cardiología en el 2021 para pacientes hospitalizados con insuficiencia cardíaca descompensada en una unidad de cuidados coronarios de un hospital de cuarto nivel de la ciudad de Bogotá.

**Materiales y métodos.:**

Estudio descriptivo de corte transversal que incluyó pacientes hospitalizados en la Unidad de Cuidados Coronarios en el Hospital San José de Bogotá, diagnóstico principal de insuficiencia cardíaca descompensada, desde septiembre de 2021 hasta enero del 2023. Se recolectaron datos de las historias de los pacientes. Se describió la adherencia a la guía en el apartado de insuficiencia cardíaca descompensada.

**Resultados.:**

Se observó una alta adherencia a los paraclínicos y prescripción de medicamentos recomendados por la guía de la Sociedad Europea de Cardiología del 2021; asimismo, baja adherencia en la solicitud de pruebas de función tiroidea, troponina y ferrocinética. Se registró adecuadamente la causa de falla cardíaca y la causa de descompensación. El motivo de descompensación más frecuente fue el síndrome coronario agudo. Dentro del perfil hemodinámico de ingreso, la mayoría presentaron Stevenson B. La adherencia farmacológica a las recomendaciones clase I demostró un alto cumplimiento en la indicación de betabloqueadores, inhibidores de la enzima convertidora de angiotensina, antagonistas del receptor de angiotensina II e inhibidores del receptor de angiotensina-neprilisina. Se registró una menor adherencia para los inhibidores del cotransportador de sodio-glucosa tipo 2 y para los antagonistas de los receptores de la aldosterona.

**Conclusiones.:**

Se observaron cifras de adherencia variables, destacando un cumplimiento satisfactorio de las recomendaciones de clase I para ciertos medicamentos y pruebas de laboratorio. Es necesario mejorar la adherencia en la solicitud de paraclínicos, especialmente en las pruebas de función tiroidea y el perfil ferrocinético.

## Abstract (en)

**Objective.:**

To assess adherence to the recommendations for the diagnosis and management of hospitalized patients with Decompensated Heart Failure issued by the European Society of Cardiology in 2021 at a Coronary Care Unit at a fourth-level hospital in the city of Bogotá.

**Materials and Methods.:**

A descriptive cross-sectional study was conducted, including hospitalized patients in the Coronary Care Unit at Hospital San José in Bogotá, with a primary diagnosis of Decompensated Heart Failure, from September 2021 to January 2023. Patient data were collected from medical records. Adherence to the Decompensated Heart Failure guidelines was described in the study.

**Results.:**

High adherence was observed for laboratory tests and medication prescriptions recommended by the 2021 European Society of Cardiology guidelines. However, there was low adherence to the request for thyroid function tests, troponin, and iron studies. The cause of heart failure and decompensation was adequately recorded. The most common cause of decompensation was acute coronary syndrome. Regarding the hemodynamic profile on admission, the majority presented as Stevenson B. Pharmacological adherence to Class I recommendations showed high compliance in prescribing beta-blockers, angiotensin-converting enzyme inhibitors, angiotensin II receptor blockers, and Angiotensin Receptor-Neprilysin Inhibitors. However, lower adherence was observed for Sodium-glucose co-transporter two inhibitors and Mineralocorticoid receptor antagonists.

**Conclusions.:**

Variable adherence rates were recorded, emphasizing satisfactory compliance with class I recommendations for certain medications and laboratory tests. It is necessary to improve adherence in the request for paraclinicals, especially in thyroid function tests and ferrokinetic profiles.

## Introducción

Las enfermedades cardiovasculares han ocupado los primeros lugares en prevalencia y morbimortalidad en las últimas décadas. Actualmente, constituyen la primera causa de mortalidad atribuible a enfermedades crónicas no transmisibles a nivel mundial según datos de la Organización Mundial de la Salud (OMS) ^1^. La insuficiencia cardíaca (IC) se manifiesta como el desenlace de múltiples enfermedades cardiovasculares cuya causa más común es la cardiopatía isquémica y es causa frecuente de ingreso a los hospitales con altas tasas de mortalidad y morbilidad ^2^^,^^3^.

A nivel mundial se ha estimado una prevalencia entre 1 a 12% ^4^ y una incidencia de 358 a 527 casos por 100 000 personas/año según los estudios ^5^, mientras que las tasas de supervivencia a 5 años se han estimado de 57% en pacientes ambulatorios y 25% para pacientes hospitalizados ^6^. En América Latina se ha estimado una incidencia de 199 casos por cada 100 000 personas/año, la mortalidad intrahospitalaria de 11,7 y de 24,5% al año de la hospitalización ^7^. En Colombia se estima una prevalencia general aproximada de 2,3%, asociado a una incidencia de dos casos por cada 1000 personas/año entre los 35 y 64 años, cifra que aumenta a 12 por cada 1000 persona/año entre los 65 y 94 años ^8^.

La prescripción adecuada y la adherencia farmacológica de pacientes con IC es fundamental para lograr un buen control de su enfermedad, previniendo las descompensaciones y hospitalizaciones relacionadas ^9^. La tasa de prescripción adecuada de medicamentos para la IC puede ser alta en contextos especializados como las clínicas de falla cardíaca, donde se ha reportado prescripciones adecuadas de 98,6% para betabloqueadores (BB) y 93,4% para antagonistas del receptor de mineralocorticoides (MRA) ^10^; sin embargo, estas tasas son menores, en otros contextos de prescripción, variando entre 33 - 79%, dependiendo del grupo farmacológico ^11^^,^^12^. En nuestra institución, un estudio en pacientes hospitalizados por medicina interna mostró un cumplimiento en la adherencia farmacológica de la guía evaluada entre 52,6 y 78,5% ^13^.

En el contexto de unidades de cuidados coronarios (UCC) no se conocen datos o estudios en la literatura que permitan establecer el nivel de cumplimiento y adherencia a las recomendaciones emitidas por guías internacionales. Por lo anterior, el objetivo de este estudio es evaluar la adherencia en las recomendaciones para el diagnóstico y manejo emitidas por la Sociedad Europea de Cardiología (SEC) en el 2021 para pacientes hospitalizados con IC descompensada (ICD) en una UCC en un hospital de cuarto nivel de la ciudad de Bogotá, efectuando una descripción detallada de la prescripción farmacológica, de laboratorios y uso de dispositivos médicos de la UCC. Se realizó la selección específica de las recomendaciones de la SEC en ICD debido a que son las adoptadas e implementadas por la UCC, teniendo estas una base científica rigurosa contando la participación de expertos en el campo, un proceso de revisión precisa, un enfoque multidisciplinario, una actualización continua, amplia aplicabilidad clínica y gran reconocimiento internacional.

## Materiales y métodos

### Diseño y población del estudio

Se realizó un estudio descriptivo de corte transversal que incluyó pacientes hospitalizados en la UCC en el Hospital San José de Bogotá, con diagnóstico principal de ICD, desde septiembre de 2021 hasta enero de 2023. Se excluyeron pacientes en estado de embarazo, pacientes que presentaban traslados o remisiones a otra dependencia hospitalaria y paciente que por cualquier motivo tengan limitaciones de las intervenciones médicas o estén en cuidados paliativos.

### Variables

Se recolectaron datos de las historias de los pacientes, incluyendo las características demográficas, fracción de eyección del ventrículo izquierdo (FEVI), la clasificación clínica de Stevenson, la escala NYHA, los antecedentes, factores de riesgo, estado de congestión, lugar de egreso, mortalidad y motivo de muerte. Se evaluó el manejo global realizado a los pacientes, en particular los siguientes aspectos: causa de descompensación, clasificación de presentación según perfil hemodinámico, intervenciones con dispositivos cardíacos e indicación de ventilación mecánica no invasiva. Se describió la adherencia a las recomendaciones emitidas por la guía de la SEC en 2021 para los apartados de solicitud de paraclínicos y tratamiento al egreso de la UCC. Los paraclínicos considerados «recomendados» por las guías incluyen el electrocardiograma (ECG), ecocardiograma, troponina, creatinina, sodio sérico, potasio sérico, porcentaje de saturación de transferrina y ferritina.

La adherencia de recomendaciones categorizadas como de clase I incluye oxígeno suplementario en pacientes con saturación parcial de oxígeno (SpO_2_) < 90% o presión arterial de oxígeno (PaO_2_) < 60 mmHg, diuréticos de asa intravenoso para pacientes con signos o síntomas de sobrecarga de líquidos, profilaxis para el tromboembolismo pulmonar en pacientes no anticoagulados sin contraindicación para anticoagulación, evaluación de signos persistentes de congestión antes del egreso de la UCC y tratamiento médico oral basado en la evidencia (clase I) para pacientes con FEVI reducida (≤40%) , y tratamiento para los pacientes con FEVI levemente reducida (41%-49%) y conservada (≥50%).

### Análisis estadístico

Se escogió la población por conveniencia durante el período de estudio. Las variables continuas se reportaron con medidas de tendencia central y dispersión utilizando mediana y rango intercuartil (RIQ) para aquellas variables con distribución no normal y media y desviación estándar (DE) para aquellas variables con distribución normal. Las variables categóricas se expresan como frecuencias absolutas y porcentajes. Para los análisis estadísticos se utilizó el *software* Stata V.18.

### Consideraciones éticas

El estudio se realizó bajo los principios de la declaración de Helsinki y fue aprobado por el Comité de Investigaciones de la Facultad de Medicina de la Fundación Universitaria de Ciencias de la Salud, con aprobación expedita con número de aprobación DI - I - 0280 - 22. Este trabajo no recibió financiación de convocatorias internas o externas.

## Resultados

**Figura 1:**
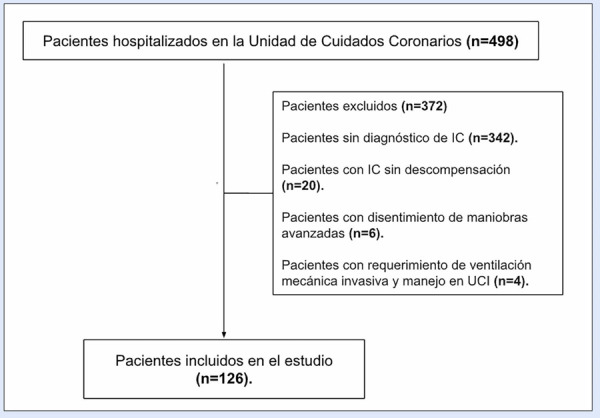
Selección de pacientes

De 498 pacientes hospitalizado en la UCC en el periodo establecido, 126 cumplieron criterios de inclusión **(**Figura 1**)**. La mediana de edad fue de 70 años (RIQ 59-77 años), 43 pacientes fueron de sexo femenino (34,1%). Se encontró una mediana de hospitalización en sala general de 11 días (RIQ: 6-20 días) y en UCC de 6 días (RIQ: 3-9 días), con una mortalidad de 6,3%. Se registró adecuadamente la causa de falla cardíaca en 108 pacientes (85,7%), y se reportó la causa de descompensación en 101 pacientes (80,2%).

IC: insuficiencia cardíaca. UCI: unidad de cuidados intensivos

El motivo de descompensación más frecuente fue el síndrome coronario agudo (n=24, 19%) y la causa más prevalente de IC fue la cardiopatía isquémica (n=47, 37,3%). Los detalles sociodemográficos y de antecedentes se indican en la Tabla 1**.** Dentro del perfil hemodinámico de ingreso, la mayoría presentaron una categoría Stevenson B (n=85, 67,5%), encontrando una frecuencia cardíaca mediana de 76 latidos por minuto (RIC=70-86) y una presión arterial sistólica de 120 mmHg (RIC=109-135). En la Tabla 2 se detallan los datos clínicos y paraclínicos al momento del ingreso.

**Tabla 1:** Características sociodemográficas de la población

Características demográficas		
Edad mediana - años (RIC)	70 (59-77)
Sexo femenino - No (%)	43 (34,1)
Antecedentes patológicos - n (%)		
Hipertensión arterial	86 (68,3)
Tabaquismo	51 (40,5)
Enfermedad coronaria	39 (31)
Diabetes mellitus	36 (28,6)
Fibrilación auricular	31 (24,6)
Valvulopatía	27 (21,4)
Enfermedad pulmonar obstructiva crónica	26 (20,6)
Enfermedad renal crónica	21 (16,7)
Síndrome de apnea-hipopnea obstructiva del sueño	14 (11,1)
Ataque cerebrovascular	9 (7,1)
Antecedentes quirúrgicos cardiovasculares - n (%)		
Cardiodesfibrilador implantable	14 (11,1)
Reemplazo valvular	12 (9,5)
Revascularización coronaria	11 (8,7)
Dispositivos de resincronización cardiaca	7 (5,6)
Marcapasos	7 (5,6)
Causa principal de la falla cardíaca - n (%)		
Isquemia miocárdica	47 (37,3)
Valvular	15 (11,9)
Dilatada Idiopática	12 (9,5)
Hipertensión arterial crónica	10 (7,9)
Arritmia cardíaca	10 (7,9)
Core pulmonar	6 (4,8)
Cardiopatía chagásica	2 (1,6)
Miocarditis	1 (0,8)
Infiltrativa	1 (0,8)
Otra	7 (5,6)
Sin registro	15 (11,9)
Causas de descompensación - n (%)	
Síndrome coronario agudo	24 (19)
Causas valvulares	19 (15,1)
Fibrilación auricular	14 (11)
Infecciones sistémicas	7 (5,6)
Pobre adherencia ambulatoria	6 (4,8)
Emergencia hipertensiva	5 (4)
Embolismo pulmonar	4 (3,2)
Otro	22 (17,5)
Sin registro	25 (19,8)

**Tabla 2:** Variables clínicas y paraclínicas al ingreso y paraclínicos

Variables clínicas al ingreso - mediana (RIC)		
Frecuencia cardíaca (latidos/minuto)	76 (70-86)
Presión arterial sistólica (mmHg)	120 (109-135)
Presión arterial diastólica (mmHg)	72 (64-79)
Peso (kg)	65 (58-76)
IMC (kg/m^2^)	25 (22-27)
Saturación de oxígeno (%)	92 (91-94)
Perfil hemodinámico acorde clasificación de Stevenson n 126 (%)	
B	85 (67,5)
C	19 (15,1)
L	3 (2,4)
Sin registro	19 (15,1)
Laboratorios al ingreso - mediana (RIC)		
Hemoglobina* (g/dL)	13,6 (2,7)
Creatinina (mg/dL)	1,1 (0,9-1,5)
Nitrógeno ureico en suero (mg/dL)	27 (19-37)
Sodio (mEq/L)	136 (134-139)
Potasio (mEq/L)	4,4 (3,9-6,3)
Hierro total (mg/dL)	66 (40-97)
Ferritina (mg/dL)	129,5 (55,6-284,5)
Saturación de transferrina (%)	21 (12-32)
Capacidad total de unión al hierro* (ug/dL)	289 (69,8)
pH	7,43 (7,39-7,46)
PCO_2_ (mmHg)	33 (29-36)
PO_2_ (mmHg)	72.5 (66-89)
Base exceso	-0,35 (-4,6-0)
Ácido láctico (mg/dL)	1,4 (1-1,8)
Dímero D (mg/dL)	1199 (480-3300)
NT-ProBNP (pg/dL)	5527 (809-16179)
Hormona estimulante de la tiroides (mUI/L)	3,4 (2,3-6)
FEVI ≤ 40 %	77 (61,1)
FEVI 41-49%	13 (10,3)
FEVI ≥ 50 %	34 (27)
FEVI no reportada	2 (1,6)

* Variables expresadas como media y desviación estándar.

IMC: índice de masa corporal. PCO_2_: presión parcial de dióxido de carbono. PO_2_: presión parcial de oxígeno. NT-ProBNP: fracción N-terminal del péptidos natriurético cerebral. FEVI: fracción de eyección del ventrículo izquierdo.

En cuanto al manejo recibido en la UCC, los fármacos más formulados fueron los diuréticos 90,5% (n=114), vasodilatadores 12,7% (n=16), vasopresores 14,3% (n=18) e inotrópicos 32,5% (n=41). Se indicó la implantación de marcapasos bicameral en un paciente y CRT-D en tres pacientes (2,4%). Se utilizó ventilación mecánica no invasiva en seis pacientes (5,6%). En cuanto al manejo al egreso de la UCC, a 51,6% de los pacientes (n=65) se formuló inhibidores del receptor de angiotensina-neprilisina (ARNI), 25,4% (n=32) antagonistas del receptor de angiotensina II (ARA II), 15,1% (n=19) inhibidores de la enzima convertidora de angiotensina (IECA), 88,9% (n=112) BB, 52,4% (n=66) MRA, 56,3% (n=71) inhibidores del cotransportador de sodio-glucosa tipo 2 (iSGLT2), y 11,1% (n=14) ivabradina.

En cuanto a la adherencia de las recomendaciones por parte del personal de salud, se registraron las siguientes cifras en la solicitud del ECG, nitrógeno ureico, electrolitos y ecocardiograma con 100% (n=126), 99,2% (n=125), 97,6% (n=123) y 95,2% (n=120), respectivamente. Solamente a la mitad de los pacientes se les midieron los valores de transferrina (n=63, 50%) y ferritina (n=64, 50,8). Desde el punto de vista farmacológico el 100% (n=126) recibió oxígeno suplementario en caso de requerirlo y el 97,6% (n=123) recibió diurético del asa. Solamente en la mitad de los casos se registró el estado de euvolemia al momento del egreso (n=62, 49,2%). La adherencia farmacológica a las recomendaciones clase I en los pacientes con IC y FEVI ≤ 40% (n=77) demostró un alto cumplimiento en la indicación de BB (n=75, 97,4%) y IECA, ARA II o ARNI (n=73, 94.8). Se registró una menor adherencia para la indicación de iSGLT2 y MRA con 80,5 (n=62) y 74% (n=57). En la Tabla 3 se registra la adherencia a las recomendaciones de manejo. 

**Tabla 3:** Adherencia a las recomendaciones de cuidado de la SEC

Adherencia a la solicitud de paraclínicos - n (%)		
Electrocardiograma	126 (100)
Nitrógeno ureico	125 (99,2)
Creatinina sérica	124 (98,4)
Sodio	123 (97,6)
Potasio	123 (97,6)
Ecocardiograma	120 (95,2)
Radiografía de tórax	111 (88,1)
Troponina	71 (56,3)
Ferritina	64 (50,8)
Transferrina	63 (50)
TSH	63 (50)
Adherencia a la prescripción de medicamentos clase I en todos los pacientes - n (%)		
Oxígeno suplementario si SpO2 < 90% o PaO2 < 60 mmHg	126 (100)
Diurético del asa endovenoso	123 (97,6)
Tromboprofilaxis	118 (93,7)
Adherencia a la prescripción de medicamentos clase I en los pacientes con IC y FEVI ≤ 40% - n (%)
BB	75 (97,4)
IECA, ARAII o ARNI	73 (94,8)
iSGLT2	62 (80,5)
MRA	57 (74)

SEC: Sociedad Española de Cardiología. TSH: hormona estimulante del tiroides. SpO_2_: saturación parcial de oxígeno. BB: betabloqueadores. IECA: inhibidores de la enzima convertidora de angiotensina. ARAII: antagonistas de los receptores de la angiotensina II. ARNI: inhibidor del receptor de angiotensina-neprilisina. iSGLT2: inhibidores del cotransportador sodio-glucosa tipo 2. MRA: antagonistas del receptor de mineralocorticoides.

## Discusión

La IC crónica es una condición que requiere hospitalizaciones frecuentes debido a episodios de descompensación aguda. Durante estas hospitalizaciones, tanto el tratamiento en el hospital como la prescripción adecuada al alta son cruciales para asegurar que los pacientes obtengan el máximo beneficio terapéutico, disminuir la tasa de reingreso hospitalario y mortalidad. En nuestro estudio se observó una alta adherencia a la mayor parte de paraclínicos solicitados y a la prescripción de medicamentos recomendados por la guía SEC 2021, aun cuando se observó baja adherencia en la solicitud de pruebas de función tiroidea y evaluación de los parámetros de la ferrocinética.

En el presente estudio se evidencio menor proporción de la enfermedad en el sexo femenino, en un 34,1%, similar a cohortes colombianas ^14^; en cuanto a causa de IC la más frecuente fue la cardiopatía isquémica, semejante a lo encontrado en cohortes internacionales, en las cuales varía entre un 40 y 67% ^11^^,^^12^. La causa de descompensación más común fue el síndrome coronario agudo seguida por causas mecánicas valvulares, datos también semejantes a los del estudio *European Society of Cardiology Heart Failure Long-Term Registry* (ESC-HF-LT), en el cual se evidenció que hasta el 68% de las causas de descompensación corresponden al síndrome coronario agudo, seguida de la valvular en un 27% ^15^. Los datos recolectados mostraron que en el 11,9% de los casos no se realizó la búsqueda de la causa basal de IC y en un 17,5% no se registró la causa de descompensación. Este resultado debería impulsar la realización de una búsqueda activa para identificar la causa principal de IC y de su descompensación en nuestra población. Es importante recalcar la importancia de indagar la causa de base y causa de descompensación de la IC con base en el acrónimo CHAMPIT (síndrome coronario agudo, hipertensión arterial, arritmias, mecánicas, embolia pulmonar, infección, taponamiento cardiaco) recomendado por la SEC, que resume las causas más frecuentes de agudización de IC.

La tasa de adherencia fue semejante a la reportada en estudios internacionales como *Therapy in outpatients with heart failure with reduced ejection fraction* (ATA) y el ESC-HF-LT ^15^^,^^16^, en donde el promedio general de adherencia a la solicitud de paraclínicos recomendados en la guía de manejo fue del 88,3%. Los resultados mostraron una adherencia alta con medicamentos que interactúan sobre el sistema renina angiotensina aldosterona (RAAS) incluyendo a los ARA II, ARNI e IECA, mayores que en cohortes de otros estudios como el ATA o el ESC-HF-LT, donde documentan una adherencia del 79 y 92%, respectivamente ^15^^,^^16^. Vale decir que la mayoría de los estudios revisados no incluyeron los ARNI debido a la guía utilizada y el año de publicación de esta.

Los BB son parte central del manejo de IC; sin embargo, su indicación en contextos agudos tiene algunas limitaciones. En nuestro estudio se encontró una adherencia de prescripción de 97,4% de estos medicamentos en pacientes con FEVI reducida, valores similares a los registrados en el estudio ATA y ESC-HF-LT. Para el caso de los MRA el porcentaje de adherencia fue menor que el de otros grupos farmacológicos; no obstante, es mayor que la reportada en otros estudios como el *Quality of Adherence to guideline recommendations for Life-saving treatment in heart failure surve* (QUALIFY), ATA, ESC-HF-LT correspondiente al 67%, 43% y 69% respectivamente ^15^^-^^17^.

En nuestro país no identificamos estudios que evaluaron adherencia a recomendaciones de guías en contexto de internación en una UCC. Algunos estudios han evaluado la adherencia al tratamiento del paciente con falla cardíaca utilizando instrumentos de evaluación multidimensionales con escalas tipo likert ^18^ para establecer la efectividad de ciertas intervenciones. Un estudio reportó una adherencia global al tratamiento farmacológico y no farmacológico de 80,1%, lo cual resulta similar a lo reportado en nuestros resultados para la adherencia a la prescripción de medicamentos acorde a las recomendaciones ^14^. El Registro Colombiano de Falla Cardíaca (RECOLFACA) ofreció datos de formulación de tratamiento farmacológico del paciente ambulatorio cuando ingresó al registro, en donde se observó una tasa de prescripción > 90% para medicamentos que bloquean el RAAS y para BB ^19^. La prescripción de estos medicamentos también es alta en estudios en donde reportan datos de tratamientos de pacientes hospitalizados por IC ^14^^,^^20^^-^^21^. En un estudio se mostró que la prescripción de MRA fue de 57% ^21^. Aunque fue un valor por debajo del obtenido en nuestro estudio, se resalta que en nuestro caso el porcentaje de prescripción para este grupo farmacológico fue el más bajo, estando por debajo del 80%, resultado consistente con el que obtuvimos en una investigación previa en nuestra institución ^13^. Este dato es importante ya que se deben estudiar en especial las causas de la baja prescripción de estos fármacos y las intervenciones que mejoren este punto de adherencia en específico. En cuanto al uso de diuréticos, estos fueron prescritos en el 90,5%, con un porcentaje de adherencia el 97,6%, similar a cohortes nacionales ^22^.

La prescripción de solicitud de paraclínicos recomendados es importante para la búsqueda tanto de etiología como la causa de la descompensación cardiaca y para evaluar la necesidad de ajuste o prescripción de nueva terapia farmacológica. Teniendo en cuenta el estado de los pacientes que se encuentran hospitalizados en el contexto de una UCC, el valor mediano encontrado de NT- PRO BNP reportado en nuestra cohorte se correlaciona con el estado de gravedad de los pacientes con ICD. Se evidenció una adherencia para la solicitud de electrocardiograma y ecocardiograma del 100 y 95,2%, respectivamente; sin embargo, se reportaron niveles bajos en toma de ferritina, transferrina y TSH con un porcentaje del 50%. En la literatura consultada no se evidencio estudios donde se reporte porcentaje de adherencia a las recomendaciones de este apartado. Este resultado evidencia el poco énfasis que se realiza en la búsqueda de comorbilidades importantes en la falla cardiaca. También se debe tener en cuenta que se trata de paraclínicos que, como en el caso del hierro, su importancia en la evaluación y manejo de la IC es más reciente.

La evaluación de la adherencia en la UCC es importante ya que los resultados pueden ser utilizados como metas de superación e impactar en la mortalidad de los pacientes. Consideramos que nuestro centro es adherente a dichas recomendaciones, sin embargo, hace falta mejorar cifras previamente mencionadas. Estos hallazgos ejercen influencia sobre la tasa de mortalidad intrahospitalaria, la cual se registró en un 6,3% de los pacientes hospitalizados, en contraste con investigaciones internacionales que reportan un porcentaje de 9,2% ^23^. El porcentaje de registro del estado de euvolemia se debe considerar un punto de mejora para el manejo de los pacientes con ICD. De acuerdo con lo comentado por Achury *et al.*, la capacitación del paciente en el manejo de su tratamiento tanto farmacológico como no farmacológico, junto con la adecuada relación médico-paciente, conducen a una adecuada adherencia al tratamiento ^24^. El apoyo psicológico resulta igualmente importante para mejorar los niveles de adherencia ambulatoria en los pacientes con IC, permitiendo empoderar a los pacientes a sus cuidadores sobre el adecuado manejo farmacológico para mejorar los desenlaces en estos pacientes ^25^.

Entre las limitaciones se encuentra que fue un estudio unicéntrico, lo cual limita su validez externa, además tuvo un componente retrospectivo que indujo a un sesgo en la recolección de los datos; sin embargo, se anota que en la UCC gran parte de la información de las historias clínicas se recolectan en formatos estandarizados, lo cual permite que haya cierto grado de homogeneidad en el registro de la información.

En conclusión, evidenciamos una adherencia variable a las indicaciones de paraclínicos y medicamentos recomendados por la guía SEC 2021. Se identificó una baja adherencia en la solicitud de pruebas de función tiroidea, troponina y ferrocinética. El registro preciso de la causa de la insuficiencia cardíaca y de su descompensación fue registrado en la gran mayoría de los pacientes. En relación con la adherencia farmacológica a las recomendaciones de clase I, se observó un alto cumplimiento en la prescripción de BB, IECA, ARA II y ARNI. No obstante, se registró una menor adherencia en la prescripción de iSGLT2 y MRA. Estos hallazgos subrayan áreas específicas que podrían beneficiarse de intervenciones para mejorar la adherencia y optimizar el manejo clínico de la insuficiencia cardíaca.

## Fuente de financiamiento:

El proyecto no requirió financiación por parte de la FUCS ni ninguna otra institución para su realización
